# Analysis of risk factors for major adverse cardiac events in patients with multiple myeloma

**DOI:** 10.3389/fcvm.2025.1633543

**Published:** 2025-10-10

**Authors:** Yu Feng, Jingjing Zhou, Shilv Chen, Shuo Li, Tianlan Li, Yan Gao, Qianqian Wang, Yujie Xu, Chunxia Mao, Shanshan Liu, Junxia Huang

**Affiliations:** Hematology Department, The Affiliated Hospital of Qingdao University, Qingdao, China

**Keywords:** multiple myeloma, major adverse cardiovascular events, ISS stage, anthracyclines, smoking, risk stratification, cardio-oncology

## Abstract

**Objective:**

To identify risk factors for major adverse cardiovascular events (MACE) in patients with multiple myeloma (MM) and to evaluate the performance of an external risk-score–based stratification.

**Methods:**

We retrospectively analyzed 162 newly diagnosed MM patients treated at Qingdao University Affiliated Hospital (2017–2023). Baseline demographics, comorbidities, laboratory and echocardiographic indices, and treatment exposures were collected. MACE (heart failure, acute coronary syndrome, malignant arrhythmias, cardiogenic shock, or cardiac sudden death) were adjudicated during therapy. Multivariable logistic regression identified independent risk factors. Progression-free survival (PFS) was compared by Kaplan–Meier analysis. An externally derived 0–4 point cardiovascular risk score was applied and patients were grouped as low (0–1), intermediate (2), or high (3–4) risk.

**Results:**

MACE occurred in 31/162 patients (19.14%). Independent risk factors included age at diagnosis (OR = 1.059 per year), cigarette smoking (OR = 3.652), anthracycline exposure (OR = 5.850), and ISS stage III (OR = 2.593; 95% CI: 1.108–6.067; all *P* < 0.05). Using the external risk score, 79, 54, and 29 patients were classified as low, intermediate, and high risk, respectively, with a stepwise rise in MACE incidence from ≈15% (low) to ≈18% (intermediate) and ≈31% (high). Discrimination of the score for MACE was modest (ROC AUC = 0.594). Patients experiencing MACE had significantly shorter PFS.

**Conclusion:**

Age, smoking, anthracycline use, and ISS stage III independently predict MACE in MM. External risk-score stratification demonstrates a clear gradient of risk but only modest discrimination, underscoring the need for prospective validation and optimization (e.g., integrating disease stage and treatment exposures). These findings support proactive cardio-oncology assessment and tailored therapy—particularly in older, smoking, ISS III, and anthracycline-treated patients.

## 1 Introduction

Multiple myeloma (MM) is a common hematologic malignancy characterized by clonal plasma-cell proliferation, multisystem involvement, and substantial impairment of quality of life and survival. Despite the advent of proteasome inhibitors, immunomodulatory drugs, monoclonal antibodies, and autologous stem-cell transplantation, MM remains incurable for most patients, and treatment-related morbidity continues to be a major clinical challenge (1). Among extra-hematologic complications, cardiovascular involvement has emerged as a key determinant of prognosis (2).

MM predominantly affects older adults; therefore, age-related cardiovascular comorbidities frequently coexist at diagnosis. In addition, disease-related factors (e.g., high tumor burden, renal dysfunction, systemic inflammation) and therapeutic exposures (e.g., anthracyclines, carfilzomib, immunomodulatory drugs, corticosteroids) can precipitate cardiovascular toxicity, leading to a broad spectrum of cardiovascular adverse events (CVAEs) and worse outcomes (3–5). CVAEs in MM encompass venous thromboembolism (VTE), arterial thromboembolism (ATE), hypertension, arrhythmias, ischemic heart disease, pulmonary hypertension, and heart failure (HF). Within this spectrum, major adverse cardiovascular events (MACE)—typically including HF, acute coronary syndrome, malignant arrhythmias, cardiogenic shock, and cardiac sudden death—are clinically meaningful composite endpoints that capture severe events with direct survival impact.

Recognition of this cardio-oncology interface has grown rapidly. The 2022 ESC Cardio-Oncology Guidelines summarize accumulating evidence that contemporary combination regimens for MM can increase the risk of serious CVAEs and recommend structured baseline risk assessment, biomarker/echocardiographic monitoring, and standardized event adjudication in clinical practice and research (6). Complementing these recommendations, a recent study from China developed and validated a prognostic risk-score model to predict CVAEs in newly diagnosed MM, integrating clinical, biomarker, and imaging variables to facilitate early risk stratification (7).

However, real-world data delineating risk factors for MACE specifically in MM, and external evaluations of risk-score performance across diverse care settings, remain limited—particularly in Chinese cohorts. To address these gaps, we conducted a single-center retrospective study to (i) identify independent predictors of MACE in newly diagnosed MM and (ii) evaluate the stratification performance of an externally derived cardiovascular risk score in our cohort. By linking disease stage, patient characteristics, and treatment exposures with hard cardiovascular endpoints, this study aims to inform proactive cardio-oncology assessment and tailored therapeutic decision-making in MM.

## 2 Materials and methods

### 2.1 Study design and setting

We conducted a retrospective, single-center cohort study at the Affiliated Hospital of Qingdao University. Consecutive patients with newly diagnosed MM between 1 January 2017 and 31 December 2023 were screened. The study was approved by the institutional Ethics Committee (QYFY-WZLL30172) and complied with the Declaration of Helsinki. Given the retrospective design and anonymized data extraction, informed consent was waived.

### 2.2 Eligibility criteria

Inclusion criteria: (i) diagnosis of MM according to the 2022 Chinese Guidelines; (ii) complete baseline clinical, laboratory, electrocardiogram (ECG)/echocardiographic, and treatment data at diagnosis; and (iii) initiation of anti-myeloma therapy with at least four completed cycles by the time of first response assessment.

Rationale: restricting analyses to patients evaluated within the early, standardized treatment window reduces heterogeneity in cumulative cardiotoxic exposure, thereby improving comparability across individuals—particularly for dose-dependent toxicities such as those associated with anthracyclines.

Exclusion criteria: (i) New York Heart Association (NYHA) class III/IV or a documented MACE within six months prior to MM diagnosis; (ii) <4 cycles of chemotherapy or no efficacy evaluation after 2–4 cycles; (iii) severe psychiatric disease precluding reliable follow-up; and (iv) incomplete records or loss to follow-up.

### 2.3 Outcomes and definitions

Patients were followed from treatment initiation until death or 31 May 2024. The primary endpoint was the occurrence of MACE, defined as any of the following: cardiac sudden death, cardiogenic shock, acute coronary syndrome, incident or worsening heart failure (HF), and malignant arrhythmias (ventricular tachycardia/fibrillation, atrial fibrillation/flutter, sinus arrest, high-grade atrioventricular block, or severe bradycardia ≤40 bpm). Progression-free survival (PFS) were assessed per standard criteria; censoring occurred at last contact. OS was not analyzed in this revision due to follow-up maturity (see Discussion).

### 2.4 Data collection and exposures

Electronic medical records were used to extract demographics (age, sex, body mass index, smoking), comorbidities (hypertension, diabetes, coronary artery disease, arrhythmia), MM characteristics [isotype, Durie–Salmon stage, International Staging System (ISS)], laboratory values at diagnosis [white blood cell (WBC), hemoglobin, lactate dehydrogenase (LDH), creatinine, estimated glomerular filtration rate (eGFR), cardiac troponin I, N-terminal pro b-type Natriuretic Peptide (NT-proBNP)], vital signs, 12-lead ECG intervals, and echocardiography [left ventricular dimensions, left ventricular mass index [LVMI], left ventricular ejection fraction[LVEF]].

Anthracycline exposure was defined as receipt of doxorubicin, epirubicin, idarubicin, or mitoxantrone at any time during the observation window (yes/no). Detailed agent-level and cumulative dose information were not systematically recorded and could not be analyzed.

### 2.5 External risk-score application

To evaluate external risk stratification, we applied a previously published 0–4-point cardiovascular risk score for newly diagnosed MM (7) to each patient according to the authors' definitions (7). Patients were categorized as low (0–1), intermediate (2), or high risk (3–4). We then compared MACE incidence across exact scores (0–4) and across risk groups (low/intermediate/high).

### 2.6 Statistical analysis

Continuous variables were summarized as mean ± SD or median (IQR) depending on distribution and compared using the t test or Wilcoxon rank-sum test, as appropriate. Categorical variables were compared using the *χ*^2^ test or Fisher's exact test. Variables with *P* < 0.10 in univariable analyses and *a priori* covariates (age, smoking, ISS stage, anthracycline exposure) were entered into a multivariable logistic regression; results are reported as odds ratios (ORs) with 95% confidence intervals (CIs) and two-sided *P* values. Model discrimination was summarized by the area under the ROC curve (AUC). Confidence intervals and formal calibration statistics for AUC were not computed due to event-count constraints. Interaction testing (age × anthracycline; age × smoking) and sensitivity models including ASCT and PI/IMiD exposures were not performed because sparse ASCT events and near-ubiquitous PI/IMiD use (≥90%), together with the limited number of MACE events, precluded stable estimation. Analyses were conducted using SPSS v26.0 (IBM) and R v4.3; a two-tailed *α* = 0.05 was considered statistically significant. Analyses were performed using SPSS v26.0 (IBM) and R v4.3 (packages pROC, ResourceSelection, survival, survminer).

## 3 Results

### 3.1 Incidence and patterns of cardiovascular events

**Figure 1:**
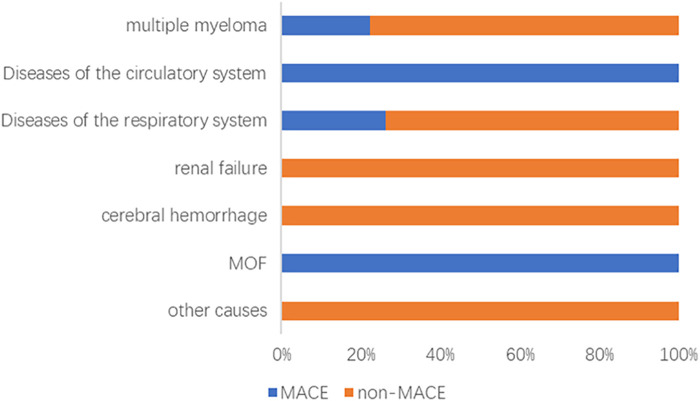
Analysis of causes of death in the MACE and non-MACE groups. MOF, multiple organ failure; MACE, major adverse cardiovascular events.

**Figure 2:**
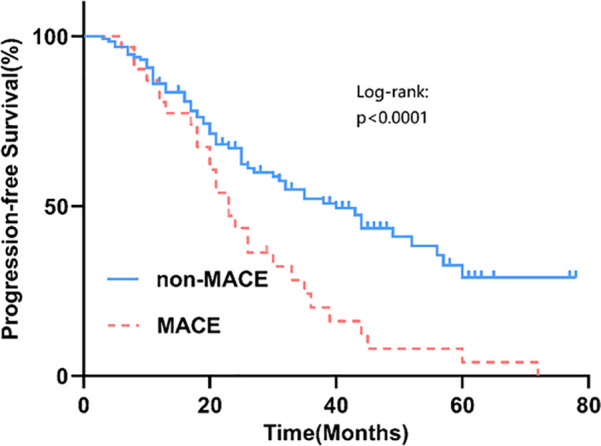
The PFS survival curves for the MACE group and the non-MACE group.

Among 162 newly diagnosed MM patients, 31 (19.14%) experienced MACE during therapy. Events comprised heart failure (*n* = 15), acute coronary syndrome (*n* = 12; 11 AMI, 1 unstable angina), and malignant arrhythmias (*n* = 4). As of 31 May 2024, 61 deaths occurred after a median follow-up of 35.5 (18.0–56.0) months; leading causes were disease progression (44.26%), respiratory diseases (29.51%), and cardiovascular events (9.84%) (Figure 1). Patients with MACE had significantly shorter PFS than those without MACE (log-rank *P* = 0.035) (Figure 2).

### 3.2 Baseline characteristics and laboratory/imaging comparisons

Compared with the non-MACE group, the MACE group was older and had higher rates of smoking, ISS stage III, and anthracycline exposure (all *P* < 0.05). Laboratory differences included higher creatinine, lower eGFR, and higher NT-proBNP in the MACE group (all *P* < 0.05). On ECG/echocardiography, QT interval and left ventricular mass index (LVMI) were greater in patients with MACE (both *P* < 0.05), whereas LVEF did not differ significantly (Tables 1–3).

**Table 1:** Comparison of demographic and general clinical data [*n* (%)].

Projects	Category	MACE (*n* = 31)	Non-MACE (*n* = 131)	*P* value
Age (years), mean ± SD	-	65.10 ± 8.43	59.25 ± 10.02	0.003*
Gender, *n* (%)				0.149
	Male	21 (67.7)	70 (53.4)	
	Female	10 (32.3)	61 (46.6)	
BMI (kg/m^2^), mean ± SD	-	23.56 ± 3.43	23.91 ± 3.18	0.585
Cigarette smoking, *n* (%)				0.012*
	Yes	14 (45.2)	30 (22.9)	
	No	17 (54.8)	101 (77.1)	
Hypertension, *n* (%)				0.731
	Yes	8 (25.8)	30 (22.9)	
	No	23 (74.2)	101 (77.1)	
CHD, *n* (%)				0.993
	Yes	3 (9.7)	10 (7.6)	
	No	28 (90.3)	121 (92.4)	
Diabetes, *n* (%)				0.999
	Yes	2 (6.5)	11 (8.4)	
	No	29 (93.5)	120 (91.6)	
Arrhythmia, *n* (%)				0.877
	Yes	3 (9.7)	9 (6.9)	
	No	28 (90.3)	122 (93.1)	
Pathological type, *n* (%)				0.545
	IgG	14 (45.2)	65 (49.6)	
	IgA	8 (25.8)	29 (22.1)	
	Light-chain	9 (29)	30 (22.9)	
	The others	0 (0)	7 (5.4)	
D-S stage, *n* (%)				0.520
	Ⅰ	2 (6.5)	4 (4.1)	
	Ⅱ	3 (9.7)	18 (13.7)	
	Ⅲ	26 (83.9)	109 (83.2)	
ISS stage, *n* (%)				0.002*
	Ⅰ∼Ⅱ	11 (35.5)	87 (66.4)	
	Ⅲ	20 (64.5)	44 (33.6)	
Therapy, *n* (%)	PIs	30 (96.8)	130 (99.2)	0.347
	IMiDs	29 (93.5)	125 (95.4)	1.000
	CTX	16 (51.6)	57 (43.5)	0.415
	Anthracyclines	11 (35.5)	16 (12.2)	0.002*
	Dara	5 (16.1)	24 (18.3)	0.775
	ASCT	2 (6.45)	26 (19.85)	0.076

BMI, body mass index; CHD, coronary heart disease; D-S, Durie-Salmon; ISS, International Staging System; PIs, proteasome inhibitors; IMiDs, immunomodulatory drugs; CTX, cyclophosphamide; Dara, daratumumab; ASCT, autologous stem cell transplantation.

*P < 0.05.

**Table 2:** Laboratory-Related data.

Projects	MACE (*n* = 31)	Non-MACE (*n* = 131)	*P* value
WBC (10^9^/L)	6.89 ± 4.01	5.77 ± 2.72	0.362
Hb (g/L)	93.39 ± 41.82	97.97 ± 27.48	0.124
LDH (mmol/L)	249.33 ± 238.67	201.13 ± 172.77	0.227
Cr (umol/L)	224.03 ± 210.32	157.00 ± 189.66	0.004*
eGFR (ml/min/1.73m^2^)	44.30 ± 27.29	60.07 ± 28.35	0.007*
cTnI (ng/ml)	0.01 ± 0.01	0.01 ± 0.02	0.137
NT-ProBNP (pg/ml)	170.19 ± 202.23	99.74 ± 138.74	0.044*

WBC, white blood cells; Hb, hemoglobin; LDH, lactic dehydrogenase; Cr, creatinine; eGFR, estimated glomerular filtration rate; cTnI, cardiac troponin I; NT-ProBNP, N-terminal pro b-type natriuretic peptide.

*P < 0.05.

**Table 3:** Comparison of baseline blood pressure, electrocardiogram, and echocardiogram.

Projects	Category	MACE (*n* = 31)	Non-MACE (*n* = 131)	*P* value
Baseline HBP				0.101
	Yes	14（45.16）	39（29.77）	
	No	17（54.84）	92（70.23）	
ECG	HR（rpm）	77.210 ± 13.22	76.21 ± 12.44	0.878
	QT interval (ms)	398.23 ± 30.72	384.76 ± 33.70	0.044*
	PR interval (ms)	163.90 ± 27.26	162.34 ± 23.36	0.501
	QRS duration (ms)	94.90 ± 9.79	92.60 ± 11.63	0.323
UCG	LVDd（cm）	4.75 ± 0.27	4.64 ± 0.35	0.125
	LVPW（cm）	0.95 ± 0.09	0.95 ± 0.10	0.829
	IVS（cm）	1.06 ± 0.11	1.04 ± 0.12	0.221
	LVEF（%）	60.61 ± 6.74	62.56 ± 2.21	0.135
	LVMI（g/m）	106.16 ± 14.79	96.64 ± 18.62	0.002*

Baseline HBP, baseline high blood pressure; ECG, electrocardiogram; HR, heart rate; UCG, ultrasound cardiogram; LVDd, left ventricular end-diastolic dimension; LVPW, left ventricular posterior wall; IVS, interventricular septum; LVEF, left ventricular ejection fraction; LVMI, left ventricular mass index.

*P < 0.05.

### 3.3 Multivariable predictors of MACE

In the multivariable logistic model (Table 4), independent risk factors for MACE were: age at diagnosis (OR: 1.059 per year; 95% CI: 1.005–1.116), cigarette smoking (OR: 3.652; 95% CI: 1.392–9.578), ISS stage III (OR: 2.593; 95% CI: 1.108–6.067), and anthracycline exposure (OR: 5.850; 95% CI: 2.035–16.81). eGFR, creatinine, NT-proBNP, QT interval, and LVMI lost significance after adjustment, consistent with collinearity with disease stage and treatment exposures (see Discussion).

**Table 4:** Multivariate logistic regression analysis.

Values	B	S.E.	Wald	*P*	OR	95%CI
Age	0.057	0.027	4.581	0.032*	1.059	1.005∼1.116
Cigarette smoking	1.295	0.492	6.929	0.008*	3.652	1.392∼9.578
ISS	0.953	0.434	4.859	0.028*	2.593	1.108∼6.067
eGFR	0.000	0.013	0.000	0.993	1.000	0.974∼1.027
Cr	0.001	0.002	0.233	0.630	1.001	0.998∼1.004
NT-proBNP	0.001	0.001	1.182	0.277	1.001	0.999∼1.004
QT interval	0.013	0.008	2.748	0.097	1.013	0.998∼1.029
LVMI	0.015	0.012	1.527	0.217	1.015	0.991∼1.041
Anthracycline drugs	1.766	0.539	10.753	0.001*	5.850	2.035∼16.81

ISS, International Staging System; eGFR, estimated glomerular filtration rate; Cr, creatinine; NT-proBNP, N-terminal pro-b-type natriuretic peptide; LVMI, left ventricular mass index.

*P < 0.05.

### 3.4 External risk-score validation

**Figure 3:**
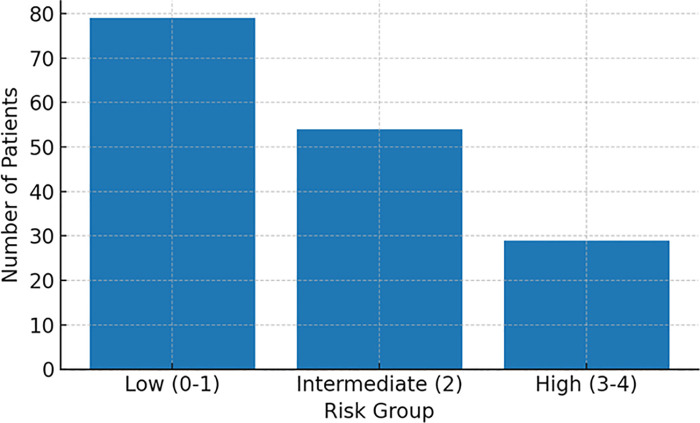
Patient count by risk group. Low, low-risk group; Intermediate, intermediate-risk group; High, high-risk group.

We applied an external 0–4-point cardiovascular risk score for newly diagnosed MM to our cohort. Grouping by established cut-offs yielded 79 low-risk (0–1), 54 intermediate-risk (2), and 29 high-risk (3–4) patients (Figure 3, Patient counts by group).

**Figure 4:**
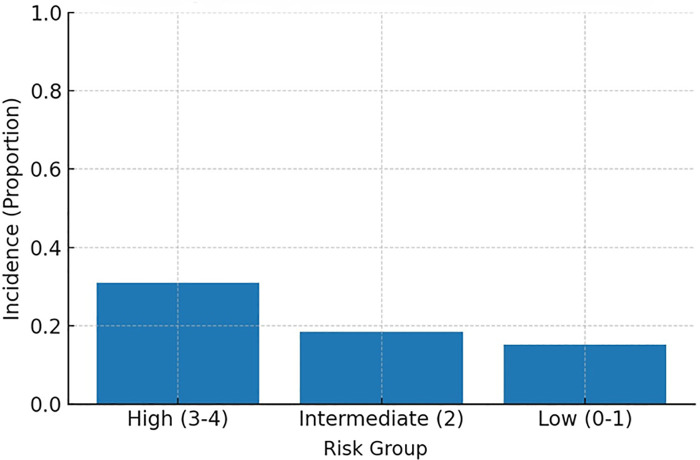
MACE incidence by risk group. High, high-risk group; Intermediate, intermediate-risk group; Low, low-risk group.

**Figure 5:**
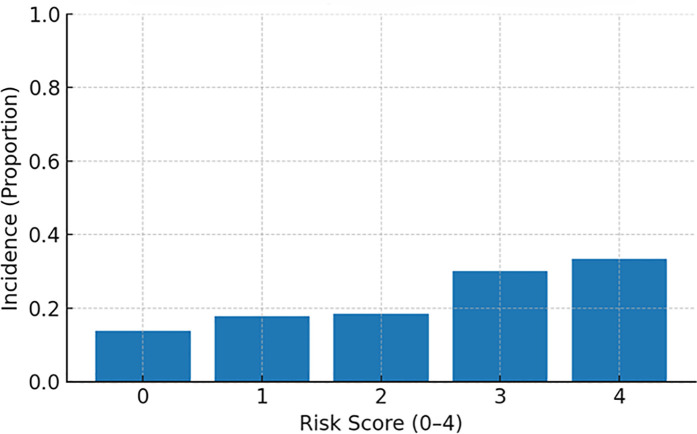
MACE incidence by exact risk score (0–4).

MACE incidence increased stepwise across categories: ∼15% (low), ∼18% (intermediate), and ∼31% (high) (Figure 4, Incidence by risk group). When examined by exact score (0–4), incidence rose monotonically from ∼14% (score 0) and ∼18% (scores 1–2) to ∼30% (score 3) and ∼33% (score 4) (Figure 5, Incidence by exact score).

**Figure 6:**
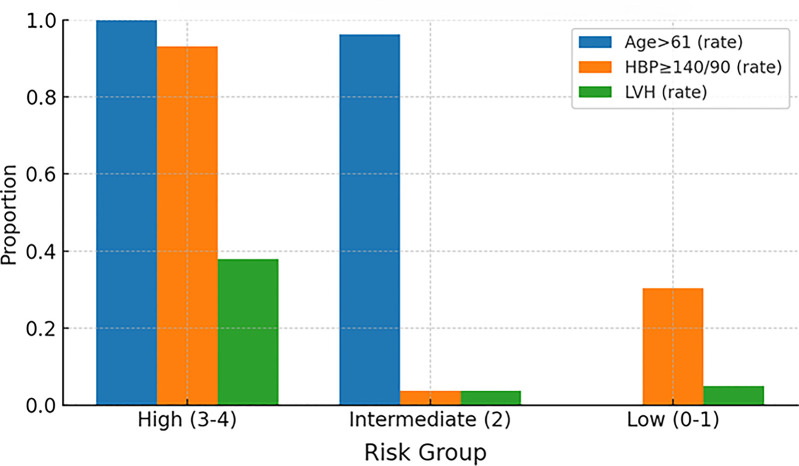
Prevalence of risk factors by group. High, high-risk group; Intermediate, intermediate-risk group; Low, low-risk group; HBP, high blood pressure; LVH, left ventricular hypertrophy.

The distribution of component risk factors across groups aligned with biological expectations: the high-risk group showed near-universal older age, a markedly higher prevalence of hypertension (≥140/90 mmHg), and a higher rate of left ventricular hypertrophy compared with the intermediate- and low-risk groups (Figure 6, Prevalence of risk factors by group).

**Figure 7:**
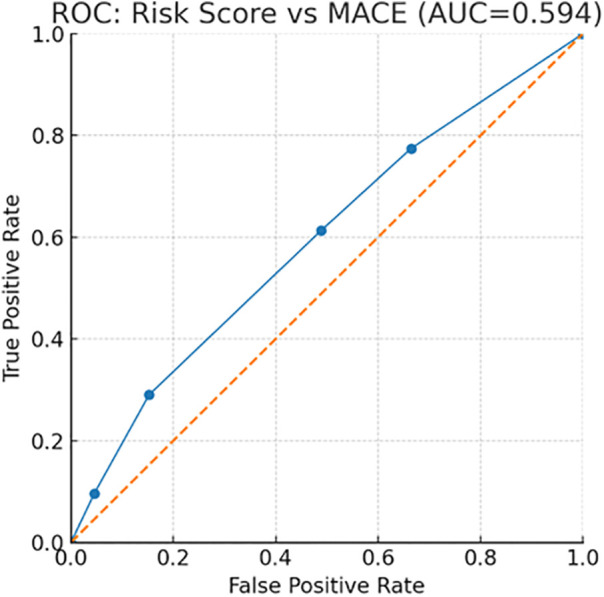
ROC curve for the risk score versus MACE.

Overall discrimination of the external score for predicting MACE in this cohort was modest with an ROC AUC = 0.594 (Figure 7, ROC curve). These findings indicate that while the score provides a clear gradient of risk, performance could be improved—potentially by incorporating ISS stage and treatment exposures (e.g., anthracyclines) identified here as independent predictors.

## 4 Discussion

Cardiovascular adverse events (CVAEs) are increasingly recognized in MM. An observational study reported CVAEs in up to 7.5% of patients with MM (5), and cardiovascular disease is a common cause of mortality in this population (8). Another study found a 12.5% cumulative incidence of cardiovascular events at initial diagnosis (9). Nevertheless, most prior work has emphasized broad CVAE composites or cardiotoxicity signals from clinical trials, with limited focus on major adverse cardiovascular events (MACE) as hard endpoints and scarce real-world data from Chinese cohorts. Moreover, externally derived cardiovascular risk scores for newly diagnosed MM have rarely been evaluated beyond their development settings. These gaps constrain risk stratification and cardio-oncology decision-making in routine practice.

In our single-center cohort of 162 newly diagnosed MM patients, 19.14% experienced MACE during therapy. Multivariable analysis identified age, smoking, anthracycline exposure, and ISS stage III as independent predictors of MACE, with ISS III showing OR = 2.593. Patients with MACE had significantly shorter PFS. Application of an external 0–4-point cardiovascular risk score demonstrated a stepwise rise in MACE across low/intermediate/high categories, but overall discrimination was modest (AUC = 0.594), indicating scope for improved real-world stratification.

Age is a shared risk factor for both cardiovascular disease and cancer (10, 11), with cardiovascular prevalence and mortality increasing steadily with aging (12). Because MM predominantly affects older adults—approximately two-thirds are ≥65 years at diagnosis (13); the international median diagnostic age is 70 years, vs. 59 years in China (14)—older patients accumulate both disease- and treatment-related vulnerabilities. Despite improvements in younger patients with novel agents, outcomes in the elderly remain inferior. For example, a recent study reported median PFS/OS of 13.6/28.9 months for patients >80 years vs. 38.3/65.6 months for those <60 years, with a decade-by-decade decline in both endpoints (15). These data underscore the heightened susceptibility of older patients to treatment-related cardiac toxicity and the need for vigilant cardiovascular surveillance in this demographic.

Smoking is another major cardiovascular risk factor. Nicotine promotes vascular remodeling through endothelial and smooth-muscle proliferation and migration (16), and large cohort data show a dose-dependent association between long-term smoking and arterial stiffness (17)—an early marker of structural/functional vascular change linked to acute coronary syndrome, stable angina, and stroke. In MM, smoking has also been associated with higher all-cause mortality (former smokers HR = 1.44; current smokers HR = 1.30) (18). Consistent with these observations, we found more smokers in the MACE than the non-MACE group, and smoking remained an independent predictor in multivariable models.

We also identified anthracycline use as an independent risk factor for MACE. Common agents include doxorubicin, mitoxantrone, epirubicin, and idarubicin. Historically, doxorubicin was frontline for MM and is still employed for refractory disease, high-risk features, or extramedullary plasmacytomas. Substantial evidence links anthracyclines to heart failure, with ventricular dysfunction reported in up to 37.5% of chemotherapy recipients (19). Proposed mechanisms include: (1) iron/free-radical hypothesis—oxidative stress from endogenous antioxidant depletion and ROS-mediated injury; (2) metabolic hypothesis—upregulated inflammatory mediators driving leukocyte chemotaxis and complement activation; (3) unified hypothesis—metabolite-induced intracellular calcium accumulation in myocytes; and (4) apoptosis hypothesis—activation of pro-apoptotic pathways in cardiomyocytes (20). Although cumulative dose data were incomplete in our retrospective dataset, the dose-dependent nature of anthracycline cardiotoxicity is well established, and the exposure signal persisted after adjustment. Anthracycline exposure could be captured only as a binary variable in this retrospective dataset; systematic agent- and dose-level information was not available, precluding dose–response analyses. Prospective standardized capture of per-cycle and cumulative doses will enable more granular risk quantification.

Importantly, ISS stage III independently associated with MACE, which is biologically plausible. ISS integrates albumin and β2-microglobulin as surrogates of disease burden and prognosis; higher stages often coincide with more severe anemia, electrolyte disturbances, and renal impairment, all linked to cardiovascular stress and events. Anemia increases cardiac workload and can precipitate ventricular dilation or heart failure, whereas renal dysfunction is closely associated with hypertension, heart failure, and coronary risk (21–23). Although direct evidence connecting ISS to cardiovascular endpoints in MM is limited, recent reports suggest higher ISS at diagnosis correlates with increased cardiovascular events, consistent with our multivariable signal and supporting the concept that disease burden *per se* contributes to MACE beyond traditional risk factors.

Regarding biomarkers, NT-proBNP differed between groups but lost significance after adjustment—most likely due to multicollinearity with disease severity (ISS) and treatment exposures. This pattern mirrors prognostic frameworks that combine biomarkers and echocardiography with clinical variables, where correlated indices may attenuate independently when modeled together.

Over the past decade, multiple cardiovascular risk-assessment models have been proposed for cancer patients receiving chemotherapy (24–26), but MM-specific tools remain limited. A recent prognostic model for MM predicted CVAEs using clinical, biomarker, and imaging variables (7). In our cohort, the external score exhibited a clear risk gradient yet only modest AUC, suggesting that model transportability would benefit from incorporating ISS and therapeutic exposures (e.g., anthracyclines) that were robust predictors here. Our findings, therefore, serve to evaluate the performance of the prior model in a real-world setting and suggest key areas for its potential refinement. By highlighting the independent predictive utility of myeloma stage and cardiotoxic treatment exposures—factors not included in the original score—our study provides a rationale for extending or recalibrating such models to improve their transportability and clinical utility.

We did not incorporate ASCT or PI/IMiD exposure in multivariable or sensitivity models. ASCT counts were small, and PI/IMiD use exceeded 90% in both groups, limiting variability and threatening model stability (quasi-separation). Our prespecified core model therefore focused on age, smoking, ISS, and anthracycline exposure.

Our study has several strengths: a clearly defined newly diagnosed cohort; systematic ascertainment of MACE as hard endpoints; multivariable modeling integrating patient, disease, and treatment factors; and external risk-score validation within the same population. Key limitations include the retrospective single-center design and modest sample size, which reduce power—especially for interaction testing—and may limit generalizability. Excluding NYHA III/IV or recent MACE at baseline probably underestimates the true incidence in higher-risk patients. Incomplete capture of cumulative anthracycline dose and some labs precluded dose–response analyses. Although we added PFS visualization, follow-up maturity and competing risks may impact long-term outcome interpretation. Finally, single-center adjudication and imaging protocols may introduce local practice effects; standardized, blinded adjudication would improve internal validity.

Implications and future directions: Clinically, our data support early cardio-oncology risk appraisal in MM and targeted surveillance/prevention for patients who are older, smoke, have ISS III, or receive anthracyclines. In line with practice recommendations, structured baseline assessment, biomarker (e.g., NT-proBNP, troponin) and echocardiographic evaluation (including LVEF and GLS where available), followed by protocolized monitoring, should be embedded in care pathways. Research priorities include multicenter studies with uniform MACE definitions, harmonized data capture, and central adjudication; prospective quantification of dose–response relationships for cardiotoxic agents; updating/recalibrating existing risk scores by adding ISS and therapeutic exposures; and interventional trials testing cardioprotective strategies (e.g., β-blockers, ACE inhibitors, anthracycline-sparing regimens or dosing modifications) in high-risk strata.

## 5 Conclusions

In this real-world cohort of newly diagnosed MM patients, we identified both disease-specific and traditional risk factors for MACE, and evaluated the performance of an external risk stratification tool. Specifically, we found that age, smoking, anthracycline exposure, and ISS stage III independently predicted MACE, and MACE was associated with worse PFS. Furthermore, while an external cardiovascular risk score stratified risk effectively, its modest discrimination (AUC = 0.594) underscores the need for model refinement that incorporates disease stage and treatment exposures. These findings support proactive cardio-oncology assessment and tailored therapeutic planning—particularly for patients at the intersection of advanced myeloma burden and cardiotoxic therapy—and provide concrete directions for standardized, multicenter, and interventional research.

## Data Availability

The original contributions presented in the study are included in the article/Supplementary Material, further inquiries can be directed to the corresponding author.
